# Review of the book: *Handicap et technologie en contextes africains* [*Disability and technology in african contexts*] by Patrick Devlieger, Jori De Coster, Lambert Nieme and Léon Mbadu-Khonde

**DOI:** 10.4102/ajod.v8i0.635

**Published:** 2019-09-15

**Authors:** Muriel Mac-Seing

**Affiliations:** 1 School of Public Health, Université de Montréal, Montreal, Quebec, Canada

In *Handicap et technologie en contextes africains* [*Disability and technology in african contexts*], Devlieger, De Coster, Nieme, Mbadu-Khonde and colleagues aim at bringing together the intersectional scholarships and testimonies of disability and technology in the sub-Saharan African context, and in particular in the cross-border Congolese settings. The book is divided into three sections. The first section illustrates the daily experience of people with disabilities and technology from various countries, beyond the description and use of assistive devices. The second section deals with the institutional contexts within which people with disabilities are evolving in terms of access to and use of technology. The third section provides different types of analysis, ranging from the interconnected representation of disability and technology to the impacts of technology on the socio-economic and cultural life of people with disabilities and their entourage.

Throughout the chapters, written either in French or in English to promote the linguistic expression of the various authors and collaborators, the book takes us through a journey on how potentially disabling situations can be metamorphosed into an emancipatory experience in life. This, provided that the agency and capabilities of individuals with disabilities are optimised and that conducive environments for their full social participation are put in place. As such, technology is understood ‘as humans using their bodies in functional ways […] is expressed in body techniques’ (Mauss 1966:20). Technology can be extended as a simple wooden walking stick to move from one point to another, a transformed tricycle to also carry commercial goods for increased financial competitiveness, a specific shunt technique to improve the quality of life of people living with hydrocephalus, or learning technical tools for children with vision and hearing impairments to become independent citizens.

For readers who are not familiar with disability-related literature, and who are inquisitive to learn more about how people with disabilities in various sub-Saharan contexts navigate in challenging attitudinal and physical environments, this book is a nice introduction to disability and will help them acquire a new perspective on the importance of the creativity often deployed by people with disabilities and their family, and of accessibility for the disabled population to work and participate in social activities like anyone else. For readers who are already cognisant of the socio-economic realities and symbolic (e.g. related to religion) complexities faced by people with disabilities, this book will also help them learn more about the powerful role played by technology in the life of people with disabilities, and through the situated positionalities of scholars and practitioners from Central and Eastern Africa, with a focus on French-speaking countries.

Furthermore, this book tells the extraordinary stories of ordinary people with disabilities, not in the classical biographical sense, but across personages and countries where common threads of lived discrimination, forced creativity, economic empowerment and social support are revealed and better apprehended. Moreover, technology is also understood as enhancing the dignity of people with disabilities, and the expression of their identity through the transformational nature technology provides them. To be fully experienced, the dignity of people with disabilities needs to be exercised through the respect of their rights by both the government and civil society organisations. Hence, the legal and institutional frameworks within which people with disabilities are evolving play a crucial role in promoting the existence of appropriate and accessible technology in Africa and elsewhere.

*Handicap et technologie en contextes africains* [*Disability and technology in african contexts*] provides us interesting perspectives and analyses, not only from researchers working on disability but also from field practitioners, clinicians, education experts and artists. It cuts across disciplines, languages and countries examining the same object of interest, that is, the intersection between disability and technology. This book contributes to the multidisciplinary and bilingual scholarship on disability by juxtaposing different linguistic, symbolic and cultural world views related to disability and technology. What I would have liked to read more is the gendered analysis of this intersection (Moodley & Graham 2015). For example, how differently (or not) is technology perceived and lived by women and men living with different types of impairments, and in various contexts of sub-Saharan Africa? I certainly look forward to reading more about the authors’ work and their further analysis of disability and technology in Africa and other regions of the world.

## Revue du livre « *Handicap et technologie en contextes africains* [*Disability and technology in african contexts*] » par Patrick Devlieger, Jori De Coster, Lambert Nieme and Léon Mbadu-Khonde

Selon Devlieger, De Coster, Nieme, Mbadu-Khonde et collègues, « *Handicap et technologie en contextes africains* **[***Disability and technology in african context***]** » a pour objectif de présenter les diverses intersections entre le handicap et la technologie, à travers différents écrits scientifiques et témoignages en Afrique subsaharienne, et particulièrement dans le contexte transfrontalier des deux rives congolaises. Le livre est divisé en trois sections. La première nous décrit l’utilisation de la technologie au sein de la vie quotidienne de personnes en situation de handicap dans divers pays, au-delà de la description et de l’utilisation des aides techniques. La seconde met de l’avant les contextes institutionnels dans lesquels celles-ci évoluent en termes d’accès et d’utilisation de la technologie. La troisième fournit différents types d’analyse des représentations imbriquées du handicap, de la technologie aux impacts de la technologie sur la vie socioéconomique et culturelle des personnes en situation de handicap et de leur entourage.

Tout au fil des chapitres écrits soit en français soit en anglais et ce, dans un souci de promouvoir l’expression linguistique des différents auteurs et collaborateurs et à l’instar d’un voyage, ce livre nous fait découvrir comment des situations potentiellement invalidantes peuvent être métamorphosées en expériences émancipatrices. Pour ce faire, l’autonomisation et les habilités des personnes en situation de handicap ainsi que les environnements favorables à leur pleine participation sociale doivent être optimisés. En tant que telle, la technologie, comprise « comme des humains utilisant leur corps de manière fonctionnelle […] est exprimée par des techniques corporelles » (Mauss 1966:20). La technologie peut ainsi être perçue par le biais d’une simple canne en bois permettant d’aller d’un endroit à un autre, d’un tricycle adapté pour transporter des biens commerciaux pour une compétitivité financière accrue, d’une technique de dérivation spécifique pour améliorer la qualité de vie des personnes atteintes d’hydrocéphalie, ou d’outils d’apprentissage destinés aux enfants ayant une incapacité visuelle ou auditive pour devenir des citoyens autonomes.

Ce livre est une belle introduction au handicap tant pour les lecteurs qui ne sont pas familiers avec la littérature sur le handicap que pour ceux qui sont avides d’apprendre davantage sur les différentes manières à travers lesquelles les personnes en situation de handicap naviguent dans le cadre d’environnements physiques et attitudinaux difficiles et ce, dans de divers contextes subsahariens d’Afrique. Il leur permettra d’acquérir de nouvelles perspectives sur l’importance de la créativité, souvent déployée par les personnes en situation de handicap et leurs familles, et de l’importance de l’accessibilité pour ces dernières afin de travailler et de participer aux activités sociales, sur un pied d’égalité avec toute autre personne. Pour les lecteurs déjà conscients des réalités socioéconomiques et des complexités symboliques, par exemple, liées à la religion, auxquelles font face les personnes en situation de handicap, ce livre leur présentera un éclairage particulier sur le rôle crucial que la technologie joue dans la vie de ces dernières, et aussi à travers les divers positionnements qu’expriment les chercheurs et les praticiens de l’Afrique centrale et de l’Afrique est, en particulier des pays francophones.

Qui plus est, ce livre nous raconte les récits extraordinaires de personnes en situation de handicap ordinaires, non pas au sens classique de leurs parcours biographiques, mais selon un fil rouge liant les différents personnages vivant dans divers pays. Cette approche nous permet de mieux saisir les discriminations vécues, la créativité nécessaire et affichée par les personnes en situation de handicap, leur *empowerment* économique et le soutien social qu’elles entretiennent. En outre, la technologie est aussi comprise comme un moyen d’améliorer la dignité des personnes en situation de handicap, et l’expression de leur dignité, grâce à la nature transformatrice que représente la technologie. Pour être pleinement incarnée, la dignité des personnes en situation de handicap s’exerce à travers le respect de leurs droits, tant par les gouvernements que par les organisations de la société civile. Les cadres juridiques et institutionnels au sein desquels elles évoluent jouent ainsi un rôle essentiel dans la promotion de l’existence de technologies appropriées et accessibles en Afrique, et ailleurs.

« *Handicap et technologie en contextes africains* **[** *Disability and technology in african context* **]** » nous offre des perspectives et des analyses intéressantes, non seulement de chercheurs œuvrant dans le domaine du handicap, mais aussi de praticiens du terrain, de cliniciens, d’experts en éducation, et même d’artistes. Cet ouvrage multidisciplinaire, bilingue et interpays, examine le même objet d’intérêt, soit l’intersection entre le handicap et la technologie. Il contribue à la recherche transdisciplinaire, en juxtaposant différentes visions du monde en ce qui a trait au handicap et à la technologie, qu’elles soient de nature linguistique, symbolique ou culturelle. En revanche, ce qui aurait été intéressant de lire est une analyse genrée de cette intersection (Moodley & Graham, 2015). Par exemple, comment la technologie est-elle perçue différemment (ou pas) par les femmes et les hommes vivant avec différents types d’incapacités et ce, dans des contextes divers d’Afrique subsaharienne? Pour finir, c’est avec impatience qu’il nous tarde de lire les futurs ouvrages et analyses des auteurs autour du handicap et de la technologie en Afrique et ailleurs dans le monde.
